# The Impact of Enhanced Recovery After Surgery on Total Joint Arthroplasty: Protocol for a Systematic Review and Meta-analysis

**DOI:** 10.2196/25581

**Published:** 2021-03-12

**Authors:** Siddharth Rele, Cade Shadbolt, Chris Schilling, Nicholas F Taylor, Michelle M Dowsey, Peter F M Choong

**Affiliations:** 1 Department of Surgery St Vincent’s Hospital University of Melbourne Melbourne Australia; 2 Melbourne Medical School University of Melbourne Melbourne Australia; 3 College of Science Health and Engineering La Trobe University Melbourne Australia; 4 Allied Health Clinical Research Office Eastern Health Melbourne Australia; 5 Department of Orthopaedics St Vincent’s Hospital Melbourne Australia

**Keywords:** enhanced recovery after surgery, total knee arthroplasty, total hip arthroplasty, systematic review, meta-analysis, postoperative outcomes, economic evaluation

## Abstract

**Background:**

The number of total joint arthroplasties (TJAs) being performed is increasing worldwide. To match this increasing demand, there has been focus on hastening patients’ recovery of function. This effort has culminated in the formulation of enhanced recovery after surgery (ERAS) strategies. However, with evolving ERAS programs and new recommendations, a review of current evidence is required to provide clinicians with up-to-date information about its effect on outcomes for TJA.

**Objective:**

The objective of this study is to assess the utility of ERAS programs on patient, health service, and economic outcomes for primary, elective total hip arthroplasty (THA) and total knee arthroplasty (TKA).

**Methods:**

A systematic search will be conducted in Medline (Ovid), EMCARE (Ovid), EMBASE (Ovid), Web of Science, CINAHL, National Health Service Economic Evaluations Database, and the Cochrane Library. Analytical, observational, and experimental designs will be included in this systematic review. Only studies including patients undergoing primary TKA and THA comparing ERAS programs with conventional surgery and postoperative care will be included. Data related to patient outcomes, health service outcomes, safety, and economic evaluation will be extracted.

**Results:**

The search terms and primary database searches have been finalized. Findings will be reported in narrative and tabular form. Where appropriate, random effects meta-analyses will be conducted for each outcome, and heterogeneity quantified with Cochran Q test and I2 statistic. Measures of effect or mean differences will be reported with 95% confidence intervals. The results of this systematic review will be disseminated in a peer-reviewed journal.

**Conclusions:**

This protocol will guide a systematic review assessing outcomes associated with ERAS surgery in primary THA and TKA.

**Trial Registration:**

Open Science Framework osf.io/y4bhs; https://osf.io/y4bhs

**International Registered Report Identifier (IRRID):**

PRR1-10.2196/25581

## Introduction

The number of total joint arthroplasty (TJA) performed worldwide is increasing [1-4]. In the United States alone, the number of total hip (THA) and total knee (TKA) arthroplasties performed each year has doubled between 2000 and 2014 [5]. With this increasing demand, reducing length of stay (LOS) has become a focus as a key hospital performance indicator [6] and a method of containing procedure-level costs [7]. Decreasing LOS for TJA [8,9] has also made way for the introduction of a strategy for outpatient surgeries [10].

Despite the importance of limiting unnecessary time in hospital following TJA, it is vital that strategies that aim to decrease LOS do not come at the expense of patients experiencing inferior postoperative outcomes. Enhanced recovery after surgery (ERAS) offers one promising approach to reducing LOS by streamlining preoperative and perioperative care [11,12]. These principles were first promulgated by Kehlet et al [13] for colorectal surgery more than 20 years ago. More recently, growing attention has been paid to whether the principles of ERAS can be employed to reduce LOS following TJA. Despite this, prior to 2020, there were no consensus statements regarding the implementation of ERAS principles in the context of arthroplasty. Since then, the ERAS society, the body responsible for proposing recommendations for ERAS protocols in surgery, has released recommendations for perioperative care after arthroplasty [14,15]. However, the proposed ERAS items highlighted in these recommendations stand in contrast to those currently published in literature—namely with the use of peripheral nerve blocks and postoperative analgesia [16].

Evidence relating to the safety and efficacy of implementing ERAS pathways is fast evolving. Recent systematic reviews by Zhu et al [11] and Deng et al [12] found ERAS programs reduced both LOS and incidence of complications in the 30 days following THA and TKA, without driving a commensurate increase in re-admission following discharge. In addition, one previous review on the cost-effectiveness of ERAS pathways found ERAS surgery to be dominant compared with conventional treatment [17]. To date, available systematic reviews have yet to explore the evidence relating to other post-acute care outcomes such as emergency department visits, postoperative primary care visits, and revision surgery. Moreover, these systematic reviews have reported only total complications and re-admissions in the 30 days following surgery, and neglected to examine outcomes over a longer period or to stratify the analysis by medical and surgical complications and re-admissions [18,19]. Furthermore, previous cost analyses have been based on small number of studies with fewer than 50 patients in each treatment arm [20,21]. Finally, the previously published systematic reviews have not assessed the impact of the number of type of items that may be combined as part of a particular ERAS program. Rather, they have focused only on comparing cohorts undergoing surgery informed by ERAS principles and those without [11,12,17].

Collectively, the limited scope of prior systematic reviews leaves us without a comprehensive picture of the potential risks associated with ERAS pathways—and with an incomplete understanding of the necessary features of a safe and effective ERAS pathway for patients undergoing arthroplasty. With these considerations in mind, the proposed systematic review and meta-analysis aims to assess the utility of ERAS programs on patient, health service, and economic outcomes for primary, elective THA, and TKA.

## Methods

### Study Reporting and Registration

This systematic review protocol will be reported according to the “Preferred Reporting Items for Systematic Review and Meta-analysis – Protocols” (PRISMA-P) [22] and the “Meta-Analysis of Observational Studies in Epidemiology” (MOOSE) [23] guidelines. In addition, this review has been registered prospectively with Open Science Framework. Any conflict between reviewers throughout the review process will be resolved through discussion; and if a consensus is not able to be reached, a third author will be consulted. The full search strategy for the primary databases can be found in Multimedia Appendix 1.

### Criteria for Inclusion

#### Type of Studies

All analytical, observational, and experimental studies will be included in this systematic review. Studies that will be excluded are descriptive studies, case reports, editorials, commentaries, qualitative studies, and literature reviews. Reference lists of relevant systematic and literature reviews will be searched to find additional studies that can be included. Only studies written in English will be included.

#### Type of Population

The population of interest is patients undergoing primary, elective THA or TKA. Studies will also be included if patients are undergoing bilateral procedures (including simultaneous and sequential procedures), as these procedure types may determine discharge destination.

Patients undergoing revision or partial arthroplasty and those undergoing arthroplasty for fractures will be excluded. Studies involving a mixed cohort will be included provided that sufficient data are present on measures of primary, elective TJA.

#### Type of Intervention

For the purposes of this review, ERAS programs will include fast-track surgery. The intervention of interest is any type of ERAS recovery program, which includes more than 1 item that is distinct from the conventional treatment program. Examples of such items include use of preoperative patient education, standardized anesthetic technique, and early and intensive mobilization.

#### Type of Comparator

The comparator of interest is conventional surgery and postsurgical pathway.

#### Outcomes of Interest

##### Primary Outcomes

These are centered around outcomes of arthroplasty and include patient outcomes, health service outcomes, and safety.

###### Patient Outcomes

Any measures of patient-reported outcome measures (eg, pain, function, global assessment, health-related quality of life) will be recorded. These will be stratified according to the timeframes examined (eg, 6 months, 1 year) up to 2 years postoperatively. In addition, measures of mobility, including, but not limited to, 6-minute walk test and walking speeding will be assessed.

###### Health Service Outcomes

These include LOS, discharge destination, duration of rehabilitation, primary care visits and emergency department visits, and re-admissions within 90 days of surgery. Re-admission will be categorized into medical and surgical causes, if possible [18]. Discharge destinations will be grouped into inpatient rehabilitation and discharge home; sensitivity analyses will be utilized to assess the impact of differing levels of postdischarge support, such as home with support, home without support.

###### Safety

This includes data on patient safety, including mortality, and any complications. All will be reported for 30 and 90 days. Complications will be grouped and organized into type of complication (eg, medical or surgical complication [18]). In addition, complications tied directly to the procedure, including surgical site infections, stiffness, manipulations under anesthesia, and revision surgery, will have no time restrictions placed.

##### Secondary Outcomes

These outcomes are centered around economic evaluation (any measure including, but not limited to, measures of cost-effectiveness, cost savings, total cost).

### Search Strategy

To ensure that a Cochrane review has not been published on the topic of impact of reducing LOS on post-acute care, the Cochrane library has been searched using the MeSH term and keyword “Arthroplasty.” This search has confirmed a Cochrane review has not been previously published on this topic.

Database searches were devised with recommendations from an external research librarian and previous literature suggesting optimal database combinations [24]. A comprehensive literature search will be performed of MEDLINE (Ovid), Web of Science, CINAHL, Emcare (Ovid), Embase (Ovid), National Health Service Economic Evaluations Database, and the Cochrane library from their inception to January 2021. In addition, Google scholar will be utilized to supplement the primary database search and reduce the risk of publication bias. Keywords and MeSH were combined with the operator “OR” for the population and intervention. The population and intervention queries were combined with the “AND” operator.

### Study Selection

Following independent screening of the first 10% of studies, the agreed eligibility criteria will be refined by discussion between the 2 reviewers (SR and CSh), as per PRISMA-P guidelines [22]. This final eligibility criteria will then be utilized to independently screen titles and abstract of identified citations by 2 reviewers. Any arising discrepancies will be resolved through discussion between reviewers.

Forward and backward citation tracking of studies for full-text screening will be used to identify any additional studies. Following this, the same 2 reviewers will undertake screening of full-text studies to determine final eligibility for inclusion. A PRISMA flow diagram will be used to report the findings of this study selection process [25]. A further full-text review will be undertaken of potentially eligible studies. Interrater reliability will be assessed and will be reported as Cohen kappa statistic [26] to inform the agreement between reviewers.

Given that retrospective studies may report on the same population within the same timeframe, care will be taken to determine if these studies are independent. In the event that multiple studies are determined to be assessing the same population, all of these studies will be treated as one, and reference to all will be made, to reduce the risk of overestimation [27]. A further critical analysis of these studies will be undertaken to highlight any discrepancies, and authors will be contacted if such discrepancies are identified.

### Data Collection and Management

The first round of deduplication will be undertaken through EndNote X9. After this, Covidence will be used to perform further deduplication, screening, and data extraction. STATA (version 16) software will be used to conduct statistical analyses.

Two reviewers will use a standardized data extraction form to extract data from each study independently. A further comparison of this, completed, data extraction form to assess consistency and accuracy will also be undertaken. Inconsistencies will be resolved with discussion with other authors. Study authors will be contacted via email if full-text copies of included studies are unattainable or clarification of methods or results is required.

The form for data extraction will follow items 1 to 16 in the “Reporting on ERAS Compliance, Outcomes, and Elements Research (RECOvER)” checklist [28] to inform details of the article, characteristics of patients and the details of the enhanced recovery program, and compliance within the program. The details of the employed enhanced recovery strategies will also be scored against the ERAS society’s recommendation.

In addition, a results summary will be recorded with total number of patients and total number of patients with outcomes; any measure of prognostic effect (including odds ratio, relative risk and hazard ratios); standardized mean difference or mean difference and 95% confidence intervals for each outcome measure as appropriate; use of univariate/multivariate analysis; and variables included in multivariate analysis. Patient-reported outcome measures will be recorded in means (SD), and any measure of economic evaluation will be recorded.

Data extraction for health economic outcomes will additionally include the following:

- Economic evaluations: economic study design, comparators, time horizon analyzed, use of discounting.
- Models: model type, model structure, assumptions, source of data (baseline and outcome of interest), model uncertainty, method of internal/external validation.
- All: costs included, source of cost data, source of utility data, quality of life instruments used, study results (currency, cost year, incremental cost effectiveness ratio).

Any missing values will be calculated provided sufficient data are available. Study authors will be contacted to obtain missing data.

### Risk of Bias Assessment

Risk of bias will be assessed by 2 reviewers through the Cochrane Risk-of-Bias 2 (RoB 2) tool [29] for randomized trials and Newcastle-Ottawa Scale (NOS) [30] for nonrandomized studies. Health economic outcomes will be assessed with the Consensus on Health Economic Criteria (CHEC) list [31], and model-based studies with the questionnaire produced by the International Society for Pharmacoeconomics and Outcomes Research (ISPOR) [32]. A summary table presenting all articles included in the review will be used to present the results of the critical appraisal. Meta-analysis will be performed if articles have adequate data for quantitative synthesis, and those with a high risk of bias will be excluded in the form of a sensitivity analysis to determine their influence on the meta-analysis [33].

### Data Synthesis and Statistical Analysis

All studies will be presented in both tabular and narrative form. A quantitative analysis of the findings from the included studies is planned for all outcomes. A random effects model will be utilized as heterogeneity is anticipated between studies [34]. Meta-analyses will be reported with a measure of effect (for dichotomous outcomes) and mean difference (for continuous outcomes). When different outcome measures are utilized for the same outcome, a standardized mean difference will be reported [35]. 95% confidence intervals will be reported for all. Care will be taken to report continuous variables separately from categorical variables.

Heterogeneity will be assessed by the Cochran *Q* test and *I*^2^ statistic [36]. Subgroup analyses with random-effects meta-analysis will be undertaken when significant heterogeneity is encountered. If heterogeneity is not resolved through this process, exclusion of any outlying studies in the form of sensitivity analysis will be considered if a clear explanation for conflicting results is found upon review of the papers in question. Results of meta-analysis, both including and excluding papers in question, will be presented and interpreted. If variation in the results cannot be accounted for, a meta-analysis will not be conducted, and results will be presented as per the “Synthesis Without Meta-analysis” (SWiM) guidelines [37]. Pooled estimates will be reported with a forest plot and 95% confidence interval.

Several subgroup analyses are planned if data permits. Studies will be grouped according to their ERAS protocol to understand the impact of different ERAS programs on outcomes. Additionally, each ERAS protocol will be scored according to the number of ERAS society recommendations implemented to understand the cumulative impact of the ERAS recommendations [14, 15]. The impact of varying levels of compliance within ERAS programs will also be evaluated through a subgroup analysis. A separate subgroup analysis for programs using a same-day discharge or outpatient surgery will also be conducted. Finally, the impact of patient-related factors, within ERAS surgery, on outcomes will also be explored.

Furthermore, it is also possible that a single comparator may not be determinable, as a significant portion of the included studies may present LOS as a continuous variable. As such, analyses will be undertaken with LOS as a continuous and categorical variable (categories for LOS will be formulated depending on the included studies). If significant differences in specific components of ERAS programs, such as the composition of spinal anesthesia, method of preoperative education, or duration of postoperative regional analgesia, are encountered between studies, sensitivity analyses will be used to assess the impact of these differences.

### Publication Bias

Strategies employed to reduce risk of publication bias include a search strategy that does not restrict gray literature, and data from unpublished work included in meta-analysis [33,38]. Furthermore, nonreporting biases will be attempted to be identified from other factors through contour-enhanced funnel plots with an appropriate test of asymmetry for meta-analyses including at least ten studies [38,39]. Any potential sources of asymmetry will be explored; and a trim and fill method will be utilized to account for the possibility of publication bias if deemed appropriate [40].

In addition, outcomes mentioned in the “Methods” section of studies will be compared with those in the results during the data extraction process. In randomized trials, the final reported outcomes will be compared with a published protocol, if available. Both reviewers will assess the completed data extraction table to determine whether certain outcomes were included by most included studies but not addressed by a few. If reporting bias is found, studies will be investigated for possible explanations, and authors may be contacted to provide clarity [33].

### Confidence in Cumulative Evidence

The Grades of Recommendation, Assessment, Development and Evaluation (GRADE) approach will be used by 2 reviewers, to independently assess the quality of cumulative evidence [41,42]. The quality of evidence will be downgraded if significant limitations are present, as evidenced from the risk of bias. In addition, evidence will be downgraded if wide confidence intervals are encountered and if statistical heterogeneity, as assessed through the *I*^2^ statistic, is over 25% [43].

## Results

A preliminary search of the primary databases has been conducted. The final database search was conducted in January 2021. This search will also be repeated prior to final publication of results. We plan on disseminating the findings of this systematic review in a peer-reviewed journal.

## Discussion

The primary focus of previous systematic reviews published on ERAS principles have relied upon authors to propose their own definitions of ERAS surgery. With the publication of the ERAS Society’s new recommendations, having objective criteria to assess these ERAS protocols allows the effects of ERAS principles to be examined with greater clarity. The results of this systematic review will help to aggregate current research and provide greater insight into the risks and benefits of reducing LOS for TJA. In addition, this review will also aid policy makers to better understand whether reductions in LOS through ERAS pathways are occurring to the detriment of the patient and health system more broadly.

## Appendix

Multimedia Appendix 1 Search strategies for primary databases.

## Abbreviations

CHEC: Consensus on Health Economic Criteria
ERAS: enhanced recovery after surgery
ISPOR: International Society for Pharmacoeconomics and Outcomes Research
LOS: length of stay
MOOSE: Meta-Analysis of Observational Studies in Epidemiology
NOS: Newcastle-Ottawa Scale
PRISMA-P: Preferred Reporting Items for Systematic Review and Meta-analysis – Protocols
RECOvER: Reporting on ERAS Compliance, Outcomes, and Elements Research
THA: total hip arthroplasty
TJA: total joint arthroplasty
TKA: total knee arthroplasty
