# Supplementary material for: The Impact of Enhanced Recovery After Surgery on Total Joint Arthroplasty: Protocol for a Systematic Review and Meta-analysis
Source: JMIR Res Protoc. 2021 Mar 12;10(3):e25581. doi: 10.2196/25581 (PMC7998324; doi:10.2196/25581)
Supplement: Multimedia Appendix 1 [file resprot_v10i3e25581_app1.docx]

**Medline (OVID)**

1. exp Arthroplasty, Replacement, Knee/ or exp Arthroplasty, Replacement, Hip/
2. (knee adj (replace* or arthroplast*)).mp.
3. (hip adj (replace* or arthroplast*)).mp.
4. 1 or 2 or 3
5. exp perioperative care/ or exp enhanced recovery after surgery/
6. enhanced recovery after surgery.mp.
7. ERAS.mp.
8. enhanced recovery pathway*.mp.
9. rapid recovery.mp.
10. fast track.mp.
11. accelerated discharge.mp.
12. accelerated care.mp.
13. early discharge.mp.
14. early rehabilitation.mp.
15. exp Ambulatory Surgical Procedures/
16. outpatient surgery.mp.
17. same day discharge.mp.
18. next day discharge.mp.
19. 5 or 6 or 7 or 8 or 9 or 10 or 11 or 12 or 13 or 14 or 15 or 16 or 17 or 18
20. 4 and 19

**Embase (OVID) / Emcare (OVID)**

1. exp knee arthroplasty/ or exp hip arthroplasty/
2. (knee adj (replace* or arthroplast*)).mp.
3. (hip adj (replace* or arthroplast*)).mp.
4. 1 or 2 or 3
5. exp perioperative period/ or exp enhanced recovery after surgery/
6. enhanced recovery after surgery.mp.
7. ERAS.mp.
8. enhanced recovery pathway*.mp.
9. rapid recovery.mp.
10. fast track.mp.
11. accelerated discharge.mp.
12. accelerated care.mp.
13. early discharge.mp.
14. early rehabilitation.mp.
15. exp ambulatory surgery/
16. outpatient surgery.mp.
17. same day discharge.mp.
18. next day discharge.mp.
19. 5 or 6 or 7 or 8 or 9 or 10 or 11 or 12 or 13 or 14 or 15 or 16 or 17 or 18
20. 4 and 19

**Cochrane**

1. knee arthroplasty or hip arthroplasty or knee replacement or hip replacement
2. MeSH descriptor: [Arthroplasty, Replacement, Hip] explode all trees
3. MeSH descriptor: [Arthroplasty, Replacement, Knee] explode all trees
4. #1 OR #2 OR #3
5. MeSH descriptor: [Enhanced Recovery After Surgery] explode all trees
6. enhanced recovery after surgery
7. ERAS
8. enhanced recovery pathway
9. fast track
10. rapid recovery
11. accelerated discharge
12. accelerated care
13. early rehabilitation
14. outpatient surgery
15. same day discharge
16. MeSH descriptor: [Perioperative Care] explode all trees
17. #5 or #6 or #7 or #8 or #9 or #10 or #11 or #12 or #13 or #14 or #15 or #16
18. #4 and #17

**NHS EED**

1. MeSH DESCRIPTOR Arthroplasty, Replacement, Knee EXPLODE ALL TREES IN NHSEED
2. MeSH DESCRIPTOR Arthroplasty, Replacement, Hip EXPLODE ALL TREES IN NHSEED
3. ((hip or knee) adj (arthroplast* or replace*)) IN NHSEED
4. ((enhanced recovery after surgery) OR (ERAS) OR (enhanced recovery pathway*)) OR ((fast track) OR (accelerated discharge) OR (early discharge)) OR ((early rehabilitation) OR (outpatient surgery)) IN NHSEED
5. MeSH DESCRIPTOR Perioperative Care EXPLODE ALL TREES IN NHSEED
6. MeSH DESCRIPTOR Ambulatory Surgical Procedures EXPLODE ALL TREES IN NHSEED
7. #1 OR #2 OR #3
8. #4 OR #5 OR #6
9. #7 AND #8

**Web of science**

1. TS=(enhanced recovery of function OR ERAS OR enhanced recovery pathway* OR fast track OR accelerated discharge OR accelerated care OR early discharge OR same-day discharge OR next-day discharge OR outpatient surgery)
2. TS=((knee OR hip) NEAR/3 (replace* OR arthroplast*))
3. #1 AND #2

**CINAHL**

1. (MH "Arthroplasty, Replacement, Knee+") OR (MH "Arthroplasty, Replacement, Hip") OR (TX ((“hip” or “knee”) n3 (“replacement” or arthroplast*)))
2. TX (“enhanced recovery of function” OR “ERAS” OR “enhanced recovery pathway*” OR “rapid recovery” OR “fast track” OR “accelerated discharge” OR “accelerated care” OR “early discharge” OR “early rehabilitation” OR “outpatient surgery” OR “same day discharge” OR “next day discharge”) OR (MH "Perioperative Care+ OR "Enhanced Recovery After Surgery")
3. S1 AND S2
